# 1,4-Bis[2-(2-pyrid­yl)-1*H*-imidazol-1-yl]butane

**DOI:** 10.1107/S1600536808036775

**Published:** 2008-11-13

**Authors:** Ke Tan, Shun-Li Li

**Affiliations:** a Biological Scientific and Technical College, Changchun University, Changchun 130022, People’s Republic of China; b Department of Chemistry, Northeast Normal University, Changchun 130024, People’s Republic of China

## Abstract

The title compound, C_20_H_20_N_6_, was isolated from dimethyl sulfoxide solution using 2-(1*H*-imidazol-2-yl)pyridine and 1,4-dichloro­butane in the presence of NaOH.

## Related literature

For the coordination capabilities and catalytic properties of the metal complexes of *N*-heterocyclic precursors, see: Chiswell *et al.* (1964 ▶); Herrmann (2002 ▶); Herrmann & Kocher (1997 ▶). For metal complexes with *N*-donor ligands, see: Carlucci *et al.* (2005 ▶);
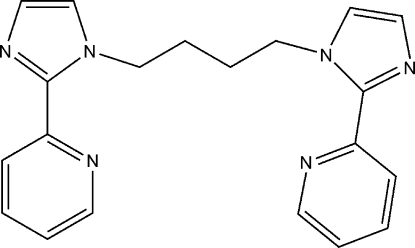


## Experimental

### 

#### Crystal data

C_20_H_20_N_6_
*M* *_r_* = 344.42
Monoclinic, 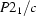

*a* = 11.0426 (10) Å
*b* = 13.4510 (12) Å
*c* = 12.7081 (11) Å
β = 111.213 (2)°
*V* = 1759.7 (3) Å^3^
*Z* = 4
Mo *K*α radiation
μ = 0.08 mm^−1^
*T* = 293 (2) K
0.43 × 0.39 × 0.36 mm

#### Data collection

Bruker SMART APEX CCD area-detector diffractometer
Absorption correction: multi-scan (*SADABS*; Sheldrick, 1996 ▶) *T*_min_ = 0.938, *T*_max_ = 0.966
10708 measured reflections
4139 independent reflections
1705 reflections with *I* > 2σ(*I*)
*R*_int_ = 0.031

#### Refinement

*R*[*F* ^2^ > 2σ(*F* ^2^)] = 0.052
*wR*(*F* ^2^) = 0.125
*S* = 1.03
4139 reflections
215 parameters
1 restraint
H-atom parameters constrained
Δρ_max_ = 0.48 e Å^−3^
Δρ_min_ = −0.47 e Å^−3^

### 

Data collection: *SMART* (Bruker, 1997 ▶); cell refinement: *SAINT* (Bruker, 1999 ▶; data reduction: *SAINT*; program(s) used to solve structure: *SHELXS97* (Sheldrick, 2008 ▶); program(s) used to refine structure: *SHELXL97* (Sheldrick, 2008 ▶); molecular graphics: *SHELXTL-Plus* (Sheldrick, 2008 ▶); software used to prepare material for publication: *SHELXL97*.

## Supplementary Material

Crystal structure: contains datablocks global, I. DOI: 10.1107/S1600536808036775/wk2095sup1.cif

Structure factors: contains datablocks I. DOI: 10.1107/S1600536808036775/wk2095Isup2.hkl

Additional supplementary materials:  crystallographic information; 3D view; checkCIF report

## Figures and Tables

**Fig. 1.**
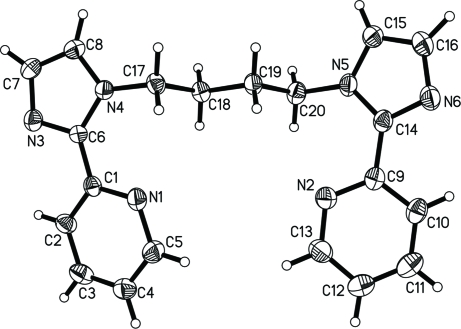
A view of the molecule of (I). Displacement ellipsoids are drawn at the 30% probability level.

## supplementary crystallographic information

### Comment

Numerous flexible or rigid N-heterocyclic precursors have been synthesized and studied because they attract considerable attention because of their diverse coordination capabilities and the important catalytic properties of their metal complexes (Herrmann, 2002; Herrmann & Kocher, 1997). A lot of metal complexes with N-donor ligands, especially ligands with imidazole-type rings separated by an aromatic spacer, have been isolated with various structures (Carlucci *et al.*, 2005). In the present work, the crystal structure of an N-donor ligand, (I), a newspacer for metal organic frameworks, is reported.

In the molecular structure of the title compound, (I), bond lengths and angles are normal. The dihedral angles between the imidazole ring and the pyridine ring in the same 2-(pyridin-2-yl)-1*H*-imidazol group are 11.6 and 37.8°, respectively. The dihedral angle between two imidazole rings in the same ligand is 13.2°. And the corresponding angle between two pyridine rings in the same ligand is 36.4°.

### Experimental

The predecessor 2-(2-pyridyl)imidazole was synthesized according to the literature (Chiswell *et al.*, 1964). A mixture of 2-(2-pyridyl)imidazole (7.25 g, 50 mmol) and NaOH (2.00 g, 50 mmol) in DMSO (20 ml) was stirred at 60°Cfor 1 h, and the 1,4-dichlorobutane(3.18 g, 25 mmol) was added. The mixture was cooled to room temperature after stirring at 60°Cfor 24 h and then poured into 200 ml of water. A yellow solid formed immediately, which was isolated by filtration in 80% yield after drying in air. Crystals suitable for X-ray diffraction were isolated from 65% ethanol.

### Refinement

All H atoms on C atoms were poisitioned geometrically and refined as idea positions, with C—H = 0.93–0.97 Å, and *U*_iso_=1.2 *U*_eq_ (C).

### Crystal data

**Table:**

C_20_H_20_N_6_	*F*_000_ = 728
*M**_r_* = 344.42	*D*_x_ = 1.300 Mg m^−^^3^
Monoclinic, *P*2_1_/*c*	Mo *K*α radiation λ = 0.71073 Å
Hall symbol: -P 2ybc	Cell parameters from 795 reflections
*a* = 11.0426 (10) Å	θ = 2.0–28.3º
*b* = 13.4510 (12) Å	µ = 0.08 mm^−^^1^
*c* = 12.7081 (11) Å	*T* = 293 (2) K
β = 111.213 (2)º	Block, colorless
*V* = 1759.7 (3) Å^3^	0.43 × 0.39 × 0.36 mm
*Z* = 4	

### Data collection

**Table:**

Bruker SMART APEX CCD area-detector diffractometer	4139 independent reflections
Radiation source: fine-focus sealed tube	1705 reflections with *I* > 2σ(*I*)
Monochromator: graphite	*R*_int_ = 0.031
*T* = 293(2) K	θ_max_ = 28.3º
ω scans	θ_min_ = 2.0º
Absorption correction: multi-scan(SADABS; Sheldrick, 1996)	*h* = −14→7
*T*_min_ = 0.938, *T*_max_ = 0.966	*k* = −17→17
10708 measured reflections	*l* = −14→16

### Refinement

**Table:**

Refinement on *F*^2^	Secondary atom site location: difference Fourier map
Least-squares matrix: full	Hydrogen site location: inferred from neighbouring sites
*R*[*F*^2^ > 2σ(*F*^2^)] = 0.053	H-atom parameters constrained
*wR*(*F*^2^) = 0.125	*w* = 1/[σ^2^(*F*_o_^2^) + (0.04*P*)^2^] where *P* = (*F*_o_^2^ + 2*F*_c_^2^)/3
*S* = 1.03	(Δ/σ)_max_ < 0.001
4139 reflections	Δρ_max_ = 0.49 e Å^−^^3^
215 parameters	Δρ_min_ = −0.47 e Å^−^^3^
1 restraint	Extinction correction: none
Primary atom site location: structure-invariant direct methods	

### Special details

**Table:**

Geometry. All e.s.d.'s (except the e.s.d. in the dihedral angle between two l.s. planes) are estimated using the full covariance matrix. The cell e.s.d.'s are taken into account individually in the estimation of e.s.d.'s in distances, angles and torsion angles; correlations between e.s.d.'s in cell parameters are only used when they are defined by crystal symmetry. An approximate (isotropic) treatment of cell e.s.d.'s is used for estimating e.s.d.'s involving l.s. planes.
Refinement. Refinement of *F*^2^ against ALL reflections. The weighted *R*-factor *wR* and goodness of fit *S* are based on *F*^2^, conventional *R*-factors *R* are based on *F*, with *F* set to zero for negative *F*^2^. The threshold expression of *F*^2^ > σ(*F*^2^) is used only for calculating *R*-factors(gt) *etc*. and is not relevant to the choice of reflections for refinement. *R*-factors based on *F*^2^ are statistically about twice as large as those based on *F*, and *R*- factors based on ALL data will be even larger.

### Fractional atomic coordinates and isotropic or equivalent isotropic displacement parameters (Å^2^)

**Table:**

	*x*	*y*	*z*	*U*_iso_*/*U*_eq_	
N1	0.34332 (12)	0.07519 (9)	0.63433 (12)	0.0497 (5)	
C1	0.39593 (14)	0.06046 (10)	0.75039 (11)	0.0417 (6)	
C2	0.46903 (15)	−0.02419 (12)	0.79408 (9)	0.0518 (7)	
H2	0.5042	−0.0341	0.8717	0.062*	
C3	0.48952 (15)	−0.09411 (9)	0.72171 (14)	0.0624 (8)	
H3	0.5384	−0.1508	0.7509	0.075*	
C4	0.43691 (16)	−0.07938 (11)	0.60565 (13)	0.0589 (7)	
H4	0.4506	−0.1262	0.5572	0.071*	
C5	0.36382 (15)	0.00527 (12)	0.56196 (8)	0.0590 (7)	
H5	0.3286	0.0151	0.4843	0.071*	
C6	0.37230 (15)	0.13348 (10)	0.81951 (12)	0.0395 (6)	
N3	0.43588 (14)	0.13294 (11)	0.93287 (12)	0.0502 (5)	
C7	0.39003 (16)	0.20944 (13)	0.97707 (11)	0.0534 (7)	
H7	0.4169	0.2262	1.0531	0.064*	
C8	0.29810 (15)	0.25725 (11)	0.89103 (13)	0.0488 (6)	
H8	0.2508	0.3126	0.8976	0.059*	
N4	0.28714 (14)	0.21030 (11)	0.79365 (11)	0.0413 (5)	
C9	−0.1363 (2)	0.11723 (18)	0.21435 (19)	0.0467 (6)	
C10	−0.1977 (3)	0.0643 (2)	0.1161 (2)	0.0572 (7)	
H10	−0.2821	0.0804	0.0697	0.069*	
C11	−0.1323 (3)	−0.0127 (2)	0.0876 (2)	0.0694 (8)	
H11	−0.1715	−0.0488	0.0215	0.083*	
C12	−0.0090 (3)	−0.03454 (19)	0.1584 (2)	0.0656 (8)	
H12	0.0381	−0.0852	0.1411	0.079*	
C13	0.0436 (3)	0.0197 (2)	0.2550 (2)	0.0633 (7)	
H13	0.1268	0.0030	0.3036	0.076*	
C14	−0.2034 (2)	0.20182 (19)	0.24123 (19)	0.0474 (6)	
C15	−0.2823 (2)	0.3070 (2)	0.3309 (2)	0.0606 (7)	
H15	−0.3017	0.3407	0.3868	0.073*	
C16	−0.3281 (3)	0.3272 (2)	0.2197 (2)	0.0679 (8)	
H16	−0.3850	0.3789	0.1863	0.081*	
C17	0.2028 (2)	0.24607 (16)	0.68080 (17)	0.0451 (6)	
H17A	0.2504	0.2412	0.6302	0.054*	
H17B	0.1837	0.3158	0.6866	0.054*	
C18	0.0760 (2)	0.19055 (17)	0.62919 (17)	0.0458 (6)	
H18A	0.0266	0.1950	0.6785	0.055*	
H18B	0.0932	0.1209	0.6205	0.055*	
C19	−0.0021 (2)	0.23502 (17)	0.51472 (17)	0.0463 (6)	
H19A	−0.0206	0.3041	0.5247	0.056*	
H19B	0.0499	0.2332	0.4674	0.056*	
C20	−0.1283 (2)	0.18114 (18)	0.45550 (17)	0.0521 (7)	
H20A	−0.1103	0.1121	0.4445	0.062*	
H20B	−0.1807	0.1827	0.5025	0.062*	
N2	−0.0161 (2)	0.09507 (15)	0.28481 (16)	0.0547 (6)	
N5	−0.20146 (18)	0.22651 (15)	0.34553 (15)	0.0499 (5)	
N6	−0.2800 (2)	0.26188 (17)	0.16254 (16)	0.0592 (6)	

### Atomic displacement parameters (Å^2^)

**Table:**

	*U*^11^	*U*^22^	*U*^33^	*U*^12^	*U*^13^	*U*^23^
N1	0.0433 (12)	0.0591 (14)	0.0435 (11)	0.0011 (11)	0.0120 (10)	0.0039 (10)
C1	0.0336 (14)	0.0477 (16)	0.0433 (14)	−0.0021 (12)	0.0134 (11)	0.0033 (12)
C2	0.0542 (17)	0.0541 (17)	0.0480 (15)	0.0130 (14)	0.0197 (13)	0.0063 (13)
C3	0.0624 (19)	0.0572 (18)	0.0724 (19)	0.0184 (15)	0.0300 (16)	0.0105 (15)
C4	0.0576 (18)	0.0644 (19)	0.0600 (18)	0.0036 (15)	0.0275 (15)	−0.0020 (14)
C5	0.0577 (18)	0.072 (2)	0.0489 (15)	−0.0003 (16)	0.0212 (14)	−0.0070 (15)
C6	0.0390 (14)	0.0402 (15)	0.0386 (14)	0.0023 (12)	0.0132 (12)	−0.0029 (11)
N3	0.0484 (13)	0.0572 (14)	0.0408 (12)	0.0002 (11)	0.0113 (10)	0.0023 (10)
C7	0.0606 (18)	0.0584 (17)	0.0407 (14)	−0.0019 (15)	0.0179 (14)	−0.0079 (13)
C8	0.0547 (17)	0.0471 (16)	0.0462 (15)	0.0000 (13)	0.0202 (13)	−0.0054 (13)
N4	0.0409 (12)	0.0417 (12)	0.0396 (11)	0.0002 (10)	0.0122 (10)	−0.0003 (9)
C9	0.0460 (17)	0.0529 (17)	0.0424 (14)	−0.0080 (14)	0.0174 (14)	0.0021 (12)
C10	0.0557 (18)	0.0694 (19)	0.0465 (16)	−0.0118 (16)	0.0184 (14)	−0.0005 (14)
C11	0.084 (2)	0.067 (2)	0.0632 (19)	−0.0193 (19)	0.0340 (19)	−0.0153 (16)
C12	0.077 (2)	0.0563 (19)	0.0702 (19)	−0.0037 (17)	0.0342 (18)	−0.0105 (16)
C13	0.0542 (18)	0.0583 (18)	0.0733 (19)	0.0057 (16)	0.0181 (16)	−0.0025 (15)
C14	0.0381 (15)	0.0605 (18)	0.0423 (15)	−0.0038 (14)	0.0128 (13)	−0.0029 (13)
C15	0.0445 (16)	0.077 (2)	0.0548 (17)	0.0102 (16)	0.0115 (14)	−0.0114 (15)
C16	0.0498 (18)	0.081 (2)	0.0626 (18)	0.0179 (16)	0.0081 (16)	0.0013 (16)
C17	0.0429 (15)	0.0441 (15)	0.0433 (13)	0.0031 (13)	0.0096 (12)	0.0067 (11)
C18	0.0421 (15)	0.0497 (15)	0.0438 (14)	0.0013 (13)	0.0132 (12)	0.0046 (11)
C19	0.0407 (14)	0.0539 (16)	0.0398 (13)	0.0023 (13)	0.0091 (12)	0.0027 (12)
C20	0.0454 (16)	0.0670 (18)	0.0435 (15)	−0.0044 (14)	0.0157 (13)	−0.0002 (12)
N2	0.0478 (14)	0.0564 (14)	0.0553 (13)	0.0004 (12)	0.0132 (12)	−0.0049 (11)
N5	0.0387 (12)	0.0689 (15)	0.0376 (12)	0.0013 (11)	0.0085 (10)	−0.0021 (11)
N6	0.0482 (14)	0.0759 (17)	0.0483 (13)	0.0105 (13)	0.0112 (12)	0.0039 (12)

### Geometric parameters (Å, °)

**Table:**

N1—C1	1.3900	C11—H11	0.9300
N1—C5	1.3900	C12—C13	1.365 (3)
C1—C2	1.3900	C12—H12	0.9300
C1—C6	1.40364	C13—N2	1.337 (3)
C2—C3	1.3900	C13—H13	0.9300
C2—H2	0.9300	C14—N6	1.325 (3)
C3—C4	1.3900	C14—N5	1.359 (3)
C3—H3	0.9300	C15—C16	1.345 (3)
C4—C5	1.3900	C15—N5	1.372 (3)
C4—H4	0.9300	C15—H15	0.9300
C5—H5	0.9300	C16—N6	1.364 (3)
C6—N3	1.3551	C16—H16	0.9300
C6—N4	1.3551	C17—C18	1.511 (3)
N3—C7	1.3551	C17—H17A	0.9700
C7—C8	1.3551	C17—H17B	0.9700
C7—H7	0.9300	C18—C19	1.520 (3)
C8—N4	1.3551	C18—H18A	0.9700
C8—H8	0.9300	C18—H18B	0.9700
N4—C17	1.480 (2)	C19—C20	1.509 (3)
C9—N2	1.338 (3)	C19—H19A	0.9700
C9—C10	1.383 (3)	C19—H19B	0.9700
C9—C14	1.464 (3)	C20—N5	1.470 (3)
C10—C11	1.385 (3)	C20—H20A	0.9700
C10—H10	0.9300	C20—H20B	0.9700
C11—C12	1.365 (3)		
			
C1—N1—C5	120.0	N2—C13—C12	124.6 (2)
N1—C1—C2	120.0	N2—C13—H13	117.7
N1—C1—C6	117.60	C12—C13—H13	117.7
C2—C1—C6	122.40	N6—C14—N5	111.6 (2)
C1—C2—C3	120.0	N6—C14—C9	122.5 (2)
C1—C2—H2	120.0	N5—C14—C9	125.9 (2)
C3—C2—H2	120.0	C16—C15—N5	106.3 (2)
C4—C3—C2	120.0	C16—C15—H15	126.9
C4—C3—H3	120.0	N5—C15—H15	126.9
C2—C3—H3	120.0	C15—C16—N6	111.0 (2)
C3—C4—C5	120.0	C15—C16—H16	124.5
C3—C4—H4	120.0	N6—C16—H16	124.5
C5—C4—H4	120.0	N4—C17—C18	114.81 (17)
C4—C5—N1	120.0	N4—C17—H17A	108.6
C4—C5—H5	120.0	C18—C17—H17A	108.6
N1—C5—H5	120.0	N4—C17—H17B	108.6
N3—C6—N4	108.0	C18—C17—H17B	108.6
N3—C6—C1	121.29	H17A—C17—H17B	107.5
N4—C6—C1	130.66	C17—C18—C19	109.66 (18)
C6—N3—C7	108.0	C17—C18—H18A	109.7
N3—C7—C8	108.0	C19—C18—H18A	109.7
N3—C7—H7	126.0	C17—C18—H18B	109.7
C8—C7—H7	126.0	C19—C18—H18B	109.7
C7—C8—N4	108.0	H18A—C18—H18B	108.2
C7—C8—H8	126.0	C20—C19—C18	112.96 (19)
N4—C8—H8	126.0	C20—C19—H19A	109.0
C6—N4—C8	108.0	C18—C19—H19A	109.0
C6—N4—C17	128.42 (14)	C20—C19—H19B	109.0
C8—N4—C17	123.39 (14)	C18—C19—H19B	109.0
N2—C9—C10	122.2 (2)	H19A—C19—H19B	107.8
N2—C9—C14	118.7 (2)	N5—C20—C19	111.43 (19)
C10—C9—C14	119.1 (2)	N5—C20—H20A	109.3
C9—C10—C11	119.3 (3)	C19—C20—H20A	109.3
C9—C10—H10	120.4	N5—C20—H20B	109.3
C11—C10—H10	120.4	C19—C20—H20B	109.3
C12—C11—C10	118.6 (3)	H20A—C20—H20B	108.0
C12—C11—H11	120.7	C13—N2—C9	116.8 (2)
C10—C11—H11	120.7	C14—N5—C15	106.33 (19)
C13—C12—C11	118.5 (3)	C14—N5—C20	129.4 (2)
C13—C12—H12	120.8	C15—N5—C20	124.2 (2)
C11—C12—H12	120.8	C14—N6—C16	104.8 (2)
			
C5—N1—C1—C2	0.0	C10—C9—C14—N5	141.1 (2)
N1—C1—C2—C3	0.0	N5—C15—C16—N6	0.5 (3)
C1—C2—C3—C4	0.0	C6—N4—C17—C18	83.6 (2)
C2—C3—C4—C5	0.0	C8—N4—C17—C18	−102.05 (19)
C3—C4—C5—N1	0.0	N4—C17—C18—C19	179.30 (18)
C1—N1—C5—C4	0.0	C17—C18—C19—C20	177.87 (19)
N4—C6—N3—C7	0.0	C18—C19—C20—N5	179.65 (19)
C6—N3—C7—C8	0.0	C12—C13—N2—C9	0.7 (4)
N3—C7—C8—N4	0.0	C10—C9—N2—C13	1.0 (3)
N3—C6—N4—C8	0.0	C14—C9—N2—C13	−177.6 (2)
N3—C6—N4—C17	175.07 (18)	N6—C14—N5—C15	0.2 (3)
C1—C6—N4—C17	−7.4 (2)	C9—C14—N5—C15	−176.9 (2)
C7—C8—N4—C6	0.0	N6—C14—N5—C20	−178.8 (2)
C7—C8—N4—C17	−175.38 (17)	C9—C14—N5—C20	4.2 (4)
N2—C9—C10—C11	−1.6 (3)	C16—C15—N5—C14	−0.4 (3)
C14—C9—C10—C11	176.9 (2)	C16—C15—N5—C20	178.6 (2)
C9—C10—C11—C12	0.6 (4)	C19—C20—N5—C14	94.6 (3)
C10—C11—C12—C13	1.0 (4)	C19—C20—N5—C15	−84.2 (3)
C11—C12—C13—N2	−1.7 (4)	N5—C14—N6—C16	0.1 (3)
N2—C9—C14—N6	142.9 (2)	C9—C14—N6—C16	177.3 (2)
C10—C9—C14—N6	−35.7 (3)	C15—C16—N6—C14	−0.4 (3)
N2—C9—C14—N5	−40.3 (3)		
